# Poor glycaemic control is associated with reduced exercise performance and oxygen economy during cardio-pulmonary exercise testing in people with type 1 diabetes

**DOI:** 10.1186/s13098-017-0294-1

**Published:** 2017-11-21

**Authors:** Othmar Moser, Max L. Eckstein, Olivia McCarthy, Rachel Deere, Stephen C. Bain, Hanne L. Haahr, Eric Zijlstra, Richard M. Bracken

**Affiliations:** 1 Diabetes Research Group, Medical School, Swansea University, SA2 8PP Swansea, UK; 2 Applied Sport, Technology, Exercise and Medicine Research Centre (A-STEM), College of Engineering, Swansea University, Fabian Way, Crymlyn Burrows, Skewen, SA1 8EN Swansea, UK; 3 Novo Nordisk A/S, Vandtårnsvej 108, 2860 Søborg, Denmark; 4 Profil, Hellenbergsstraße 9, 41460 Neuss, Germany

**Keywords:** Glycaemic control, Exercise performance, Oxygen economy, Type 1 diabetes, Heart rate turn point

## Abstract

**Background:**

To explore the impact of glycaemic control (HbA_1c_) on functional capacity during cardio-pulmonary exercise testing in people with type 1 diabetes.

**Methods:**

Sixty-four individuals with type 1 diabetes (age: 34 ± 8 years; 13 females, HbA_1c_: 7.8 ± 1% (62 ± 13 mmol/mol), duration of diabetes: 17 ± 9 years) performed a cardio-pulmonary cycle ergometer exercise test until volitional exhaustion. Stepwise linear regression was used to explore relationships between HbA_1c_ and cardio-respiratory data with p ≤ 0.05. Furthermore, participants were divided into quartiles based on HbA_1c_ levels and cardio-respiratory data were analysed by one-way ANOVA. Multiple regression analysis was performed to explore the relationships between changes in time to exhaustion and cardio-respiratory data. Data were adjusted for confounder.

**Results:**

HbA_1c_ was related to time to exhaustion and oxygen consumption at the power output elicited at the sub-maximal threshold of the heart rate turn point (r = 0.47, R^2^ = 0.22, p = 0.03). Significant differences were found at time to exhaustion between Q*I* vs. Q*IV* and at oxygen consumption at the power output elicited at the heart rate turn point between Q*I* vs. Q*II* and Q*I* vs. Q*IV* (p < 0.05). Changes in oxygen uptake, power output and in oxygen consumption at the power output elicited at the heart rate turn point and at maximum power output explained 55% of the variance in time to exhaustion (*r* = 0.74, R^2^ = 0.55, p < 0.01).

**Conclusions:**

Poor glycaemic control is related to less economical use of oxygen at sub-maximal work rates and an earlier time to exhaustion during cardio-pulmonary exercise testing. However, exercise training could have the same potential to counteract the influence of poor glycaemic control on functional capacity.

*Trial registration* NCT01704417. Date of registration: October 11, 2012

## Introduction

Type 1 diabetes (T1D) is associated with an increased risk of cardio-vascular disease (CVD) compared to people without diabetes. Although regular physical activity is encouraged as a cornerstone of good diabetes management [1, 2], physical inactivity rates remain high [3]. Patients often cite low functional capacity and loss of metabolic control (short-term and/or long term glycaemic disturbances) as barriers to beginning or maintaining regular physical activity [4].

Functional capacity, defined as the ability to perform aerobic work during maximal exercise testing can be assessed by means of cardio-pulmonary exercise (CPX) testing. This non-invasive, sensitive test provides an assessment of integrative responses of cardiovascular, pulmonary and musculoskeletal systems across a sub-maximal to maximal continuum and can be utilised to identify fatigue of cardiovascular or respiratory origin [5]. Most reported studies in people with T1D have assessed maximum cardio-pulmonary values such as the peak oxygen uptake (VO_2peak_) [6–8]. VO_2peak_ is the highest rate of oxygen that can be consumed during exercise involving the majority of muscle mass at sea-level and is defined by the Fick equation as the product of cardiac output and arteriovenous oxygen difference [5]. Achieving a true VO_2peak_ requires strong motivation and physical fitness, but for people with T1D with ‘low exercise tolerance’ the perception of exercise-induced pain may encourage premature test termination. In addition, the presence of diabetes complications such as neuropathy, nephropathy and underlying CVD may exacerbate pain or damage during exercise and reduce the validity of CPX testing in detailing accurate maximum cardio-respiratory parameters e.g. VO_2_ plateau, maximum heart rate (HR_max_).

Most activities in daily life are performed at low-to moderate intensity and do not require maximum cardio-respiratory effort. It may be more relevant in people with T1D to explore cardio-respiratory data obtained during sub-maximal stages of CPX testing. The rate of oxygen use at sub-maximal exercise intensities provides an indication of the economy of use of oxygen for an individual to the work rate and has been shown to predict endurance performance in healthy individuals [9]. Furthermore, identification of the sub-maximal work rate at which there is an increase in use of non-oxidative fuel sources (i.e. cellular glycolysis) results in a greater production of carbon dioxide (CO_2_), pyruvate and lactic acid fermentation. Identification of such ‘thresholds’ involving ventilation, heart rate and/or blood lactate have been studied as sub-maximal predictors of endurance capacity in healthy cohorts and in people with chronic disease [5]. As an example the heart rate turn point (HRTP), which is based on findings from Conconi et al., was significantly associated with the second lactate threshold [10–13]. This heart rate derived threshold is defined as the intersection of two regression lines of the heart rate to performance curve between early stages of CPX testing [peri-first lactate turn point (LTP_1_)] and maximum power output (P_max_), determined from a second-degree polynomial representation satisfying the condition of least error squares [14]. However, there is a lack of information about their use in people with T1D in predicting functional capacity.

Lower maximum cardio-respiratory variables have been reported in participants with T1D compared to healthy individuals in some studies but not in others [8, 15]. A review by Baldi et al. [16] shed some light on the influence of glycaemic control within people with T1D and when compared to healthy individuals. Patients with poor glycaemic control demonstrated a lower peak functional capacity than those patients with good glycaemic control. No information currently exists on the influence of glycaemic control on sub-maximal cardio-respiratory parameters obtained from a CPX test. Such information might reveal additional insights on the impact of glycaemia on the functioning of the cardio-vascular and respiratory system and provide further support for the use of moderate intensity exercise tests that reduce stress on the patient.

The aim of this study was to examine the relationship of glycaemic control to sub-maximal and maximum cardio-pulmonary markers obtained during CPX testing in people with T1D.

## Materials and methods

### Participant characteristics

Adults (aged 18–45 years, both inclusive) with T1D eligible for the study had a body mass index (BMI) of 18–27 kg/m^2^, glycated haemoglobin (HbA_1c_) level ≤ 9.5% (80 mmol/mol) and were performing regular physical cardiorespiratory exercise during the last 3 months before screening. Exclusion criteria included cancer, cardiac diseases, supine blood pressure outside the range 90–140 mmHg for systolic blood pressure or 50–90 mmHg for diastolic blood pressure, recurrent severe hyperglycaemia or hypoglycaemia unawareness and smoking [17]. Sixty-four people with T1D were included for analyses (Table 1). Data were extracted from a clinical trial (NCT01704417) [17].

**Table 1:** Participant characteristics given as mean ± SD and percentage (%)

Characteristic	Total (n = 64)
Age (years)	34 ± 8
Gender	
Female (n; %)	13 (20)
Male (n; %)	51 (80)
Body mass index (kg/m^2^)	24 ± 2
Blood pressure (mmHg)	124 ± 17/79 ± 12
Resting heart rate (b/min)	81 ± 12
Duration of diabetes (years)	17 ± 9
HbA_1c_ [% (mmol/mol)]	7.8 ± 1 (62 ± 13)
Total daily dose of insulin (U)	51 ± 15
Type of therapy	
Multiple daily injections (n; %)	47 (78)
Insulin pump therapy (n; %)	17 (22)
Co-morbidities	14
Arterial hypertension	6
Hypothyroidism	5
Hypercholesterolemia	2
Hashimoto thyroiditis	1
Medication other than insulin	
ACE inhibitor	6
Levothyroxine	6
Statin	2
Diuretic medication	1
Calcium channel blocker	1
Physical activity assessed via IPAQ (MET min week)	3086 ± 2736

### Study procedures

After the assessment of eligibility, patients were asked to fill in the International Physical Activity Questionnaire (IPAQ) to assess physical activity (MET min/week). Patients characteristics, medical history and medications were documented in a case report form (CRF). Afterwards, HbA1c was measured via a venous blood sample collected from the antecubital vein (Automated Glycohemoglobin Analyzer HLC-723G8, Tosoh Europe N.V, Belgium). Immediately before and after CPX testing, venous blood was collected to analyse blood glucose concentration to ensure euglycaemia during CPX testing (Super GL Glucose Analyzer, Dr. Müller Gerätebau GmbH, Germany). If pre-exercise venous blood glucose concentration was below 4.4 mmol/l carbohydrates were given (15–30 g) and if blood glucose concentration was above 13.9 mmol/l a small bolus correction dose was administered. No hypo- (< 3.9 mmol/l) or severe hyperglycaemia (> 19.4 mmol/l) occurred before or during CPX testing. The timing of bolus insulin injection was not exactly pre-defined, but participants were told to avoid the peak action of bolus insulin during CPX testing (this means avoiding bolus insulin injections less than 120 min prior to the start of CPX testing). Participants performed a CPX test until volitional exhaustion on a cycle ergometer (Ergospirometer PowerCube^®^-Ergo, Ganshorn Medizin Electronic, GER). Participants sat quietly on the cycle ergometer for 3 min (0 W) before they started the warm-up period of 3 min cycling at a workload of 30 W for females and 40 W for males. Then, the workload was increased by 30 W for females and 40 W for males every 3 min until maximum volitional exhaustion. Finally, a cool-down period was performed for 1 min.

### Measurements

Pulmonary gas exchange variables were collected continuously by breath-by-breath measurement and then averaged over 10 s. VO_2peak_ was defined as the 1 min average in oxygen (O_2_) consumption during the highest work rate. Heart rate and blood pressure were measured continuously via a 12-lead electrocardiogram and an automatic sphygmomanometer (Ergospirometer PowerCube^®^-Ergo, Ganshorn Medizin Electronic, GER).

The non-invasive anaerobic threshold was defined by the HRTP [18]. HRTP was demarcated as the intersection of two regression lines of the heart rate to performance curve between post-warm-up and maximum power output (P_max_), determined from the second-degree polynomial representation satisfying the condition of least error squares [14]. Additionally, the second ventilatory threshold (VT_2_) was determined by means of the ventilation/carbon dioxide (VE/VCO_2_) slope [19] to control for the accuracy of HRTP.

### Statistical analysis

Data (10 s average) were expressed as absolute values and relative to maximum physiological variables and P_max_. Data were tested for distribution via Shapiro-Wilks normality test and non-normal distributed data were log transformed. Stepwise linear regression was used to explore relationships between glycaemic control (HbA_1c_) and CPX obtained cardio-respiratory data and performance markers with p ≤ 0.05. Data were adjusted for sex, age, BMI, blood glucose concentration at the start of CPX testing and duration of diabetes. Post hoc power analysis for the primary outcome [stepwise linear regression: dependent variable HbA_1c_ levels, independent variables time to exhaustion (Time_max_) and oxygen economy at HRTP] resulted in a power (1-beta error probability) of 0.96.

Participants were divided into quartiles (Q) based on HbA_1c_ levels, and respective sub-maximal and maximal CPX derived cardio-respiratory data and performance markers were analysed by one-way analysis of variance (ANOVA) followed by a fishers least significant difference multiple comparison post hoc test (LSD). Multiple regression analysis was performed to explore relationships between changes in Time_max_ and independent variables, VO_2peak_ and oxygen uptake at the heart rate turn point (VO_2HRTP_), body mass adjusted values of P_max_ and power output at the heart rate turn point (P_HRTP_) as well as oxygen economy at P_max_ [VO_2peak_/P_max_ (ml/min/W)] and at HRTP [VO_2HRTP_/P_HRTP_ (ml/min/W)]. All statistics were performed with a standard software package of SPSS software version 22 (IBM Corporation, USA) and Prism Software version 7.0 (GraphPad, USA).

## Results

### Exercise performance data

Maximum physiological parameters were found at HR_max_ of 185 ± 11 b/min, VO_2peak_ 37 ± 5 ml/kg/min, respiratory exchange ratio (RER) 1.22 ± 0.09 and P_max_ 231 ± 47 W. No significant differences were found between the HRTP and the VT_2_ as well as for the comparison of pre- and post-exercise blood glucose concentration as given in Table 2.

**Table 2:** Comparison of the anaerobic thresholds derived from HRTP and VT_2_ as well as pre- and post-exercise blood glucose levels

	HRTP	VT_2_	p value
VO_2_ (l/min)	2.09 ± 0.50	2.17 ± 0.50	0.41
HR (b/min)	158 ± 14	157 ± 17	0.63
P (W)	169 ± 39	172 ± 38	0.45
VE (l/min)	55 ± 13	58 ± 14	0.26

	Pre-exercise	Post-exercise	p value
BG (mmol/l)	9.3 ± 3.4	10 ± 3.2	0.06

Results are given as mean ± SD

### Glycaemic control and functional capacity

**Fig. 1:**
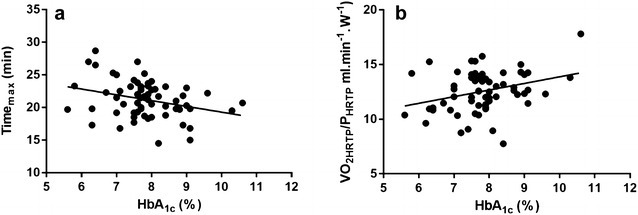
Relationships between HbA_1c_ and **a** Time_max_ and **b** VO_2HRTP_/P_HRTP_, n = 64

As shown in Fig. 1, sex-, age-, BMI-, blood glucose concentration at the start of CPX testing- and duration of diabetes-adjusted stepwise linear regression model revealed that HbA_1c_ was related to Time_max_ and oxygen consumption at the power output elicited at the sub-maximal threshold of the heart rate turn point (VO_2HRTP_/P_HRTP_) (r = 0.47, R^2^ = 0.22, p = 0.03).

### Grouped HbA_1c_ levels and functional capacity

Grouping participants based on quartiles of glycaemic control resulted in HbA_1c_ levels of 6.7 ± 0.5% (49 ± 6 mmol/mol) for quartile *I*, 7.6 ± 0.1% (60 ± 1 mmol/mol) for quartile *II*, 8.0 ± 0.1% (63 ± 1 mmol/mol) for quartile *III* and 9.1 ± 0.6% (76 ± 7 mmol/mol) for quartile *IV* (p < 0.01). No significant differences were found for physical activity (p = 0.68), resting HR (p = 0.42), systolic blood pressure (p = 0.18) and diastolic blood pressure (p = 0.83) between groups.

**Fig. 2:**
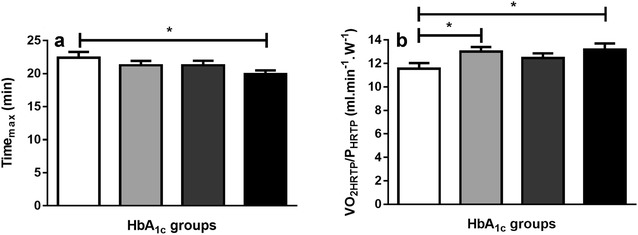
HbA_1c_ quartiles for **a** Time_max_ and **b** VO_2HRTP_/P_HRTP_

Significant differences were found at Time_max_ between Q*I* vs. Q*IV* (mean difference 2.5 ± 1.0 min, p = 0.02) and at VO_2HRTP_/P_HRTP_ between Q*I* vs. Q*II* (− 1.5 ± 0.6 ml/min/W, p = 0.02) and Q*I* vs Q*IV* (− 1.6 ± 0.71 ml/min/W, p = 0.01) (Fig. 2).

White bar = Q*I* (HbA_1c_ 6. ± 0.5%; 4 ± 6 mmol/mol), bright-grey bar = Q*II* (HbA_1c_ 7. ± 0.1%; 60 ± 1 mmol/mol), dark-grey bar = Q*III* (HbA_1c_ 8. ± 0.1%; 6 ± 1 mmol/mol) and black bar = Q*IV* (HbA_1c_ 9.1 ± 0.6%; 7 ± 7 mmol/mol). Values are given as mean and SD. “*” represents p ≤ 0.05.

### Relationships of time to exhaustion and cardio-pulmonary responses during CPX testing

Multiple regression analysis revealed that changes in VO_2peak_, VO_2HRTP_, P_max_, P_HRTP_, VO_2*HRTP*_ */*P_HRTP_ as well as VO_2peak_/P_max_ constitute independent predictors of Time_max_ (*r* = 0.74, p < 0.01) and those variables could explain 55% of the alteration in Time_max_.

## Discussion

This study demonstrated an important relationship between glycaemic control and the cardio-respiratory responses to CPX testing in people with T1D. Exercise economy is defined as the rate of oxygen use at a given workload. We found that individuals with poorer glycaemic control (higher HbA_1c_ values) displayed a higher rate of O_2_ use at a sub-maximal work rate (HRTP) and earlier Time_max_. This result is confirmed by the findings from Tagougui et al. in which the increase in deoxyhemoglobin (reflection of relative tissue deoxygenation) in the vastus lateralis was blunted in patients with T1D and with poor glycaemic control during CPX testing [20]. Interestingly, the maximum oxygen consumption was negatively correlated with changes in deoxyhemoglobin.

Our findings are in contrast to recent studies evaluating the association between glycaemic control and functional capacity [6, 8]. Stubbe et al. could not find an association between HbA_1c_ levels and the O_2_ uptake at the anaerobic lactate threshold [8]. This contrast in comparison to our results might explained by lower and more homogeneous HbA_1c_ levels as observed in our cohort. The variation in glycaemic control and its upper and lower extremes clearly contributed to the association between HbA_1c_ levels and rates of O_2_ use at the HRTP. Turinese et al. also did not find significant relationships between the glycaemic control and CPX derived markers [6]. However, the lack of associations might be originated by the low number of participants (N = 17).

There may be several postulated reasons for our findings. During exercise, skeletal muscle consumes most of the inhaled O_2_ and a greater O_2_ cost for a given exercise intensity might indicate fibre atrophy and/or morphological abnormalities in the mitochondria [21]. Reduced skeletal muscle mitochondrial ATP production rates have also been associated with poor glycaemic control [22]. Furthermore, the capillary density surrounding skeletal muscle has been shown to be lower in individuals with T1D [23]. A reduced oxidative capacity within prime mover muscles increases dependence on supporting muscles, and increases the overall oxygen cost of the activity for a given workload. Furthermore, an earlier limit on O_2_ use within a muscle shifts energy metabolism towards non-oxidative glycolysis and an earlier lowered pH [24]. Alternatively, oxygen supply systems may be compromised more in individuals with poorer glycaemic control. Red blood cell dynamics have been shown to be altered in T1D rats [25] with velocity and flux reduced in comparison to non-diabetics, albeit with a similar haematocrit.

People with T1D may display cardiomyopathy and this seems dependent on the HbA_1c_ value which has been hypothesised to alter cardiac structure, e.g. increased left ventricular wall thickness and mass, and impaired diastolic function. Interestingly, poor glycaemic control was associated with reduced stroke volume and cardiac output in athletes with T1D compared with non-diabetic individuals, despite an equivalent amount of training [26]. This reduction in stroke volume might be caused by earlier diastolic dysfunction, which reduces the atrioventricular pressure gradient and causes early diastolic left ventricular filling [16].

The decreased economy at early stages during CPX testing translated to shorter exercise test duration. Indeed, approximately 55% of the shorter total exercise time could be accounted for lowered O_2_ uptake, decreased power output and less economical use of O_2_ for both at the HRTP and maximum power. Taken this into account, it might be that regular exercise training, which increases these physiological parameters, might have similar potential to neutralise the negative influence of glycaemic control on functional capacity [27] and even on cardiovascular autonomic regulation [28].

This study is limited by the heterogeneity and the wide range in the duration of diabetes, age, blood glucose concentration at the start of CPX testing and sex distribution. However, we addressed this limitation as we have adjusted for these factors. Additionally, it might be that different levels of blood glucose influenced catecholamine response which in turn altered cardiac function during CPX testing.

## Conclusions

In conclusion, in this well-characterised study, individuals with T1D and poorer glycaemic control displayed less economical use of oxygen at sub-maximal work rates and an earlier time to exhaustion during CPX testing. Nevertheless, exercise training could have the same potential to counteract the influence of poor glycaemic control on functional capacity.

## Abbreviations

T1D: type 1 diabetes
CVD: cardio-vascular disease
CPX: cardio-pulmonary exercise
VO_2peak_: peak oxygen uptake
HR_max_: maximum heart rate
CO_2_: carbon dioxide
HRTP: heart rate turn point
LTP_1_: first lactate turn point
P_max_: maximum power output
IPAQ: International Physical Activity Questionnaire
VT2: second ventilatory
VE/VCO_2_ slope: ventilation/carbon dioxide slope
BMI: body mass index
HbA_1c_: glycated haemoglobin
O_2_: oxygen
Q: quartiles
ANOVA: analysis of variance
LSD: fishers least significant difference multiple comparison post hoc test
Time_max_: time to exhaustion
VO_2HRTP_: oxygen uptake at the heart rate turn point
P_HRTP_: power output at the heart rate turn point
RER: respiratory exchange ratio
HR_HRTP_: heart rate at the heart rate turn point
